# A Rare Presentation of Gastric Carcinoma With Gastric Perforation and Septic Shock

**DOI:** 10.7759/cureus.18657

**Published:** 2021-10-11

**Authors:** Reem Moala AlHazmi, Dunya Nasrallah Alfaraj, Shaykhah Nasser AlNaimi, Sarah Mohammed AlQahtani, Mashael Hamed AlJuwayed, Hazem Mohammed Zakriea, Mohammed S Foula

**Affiliations:** 1 College of Medicine, Imam Abdulrahman Bin Faisal University, King Fahd University Hospital, Dammam, SAU; 2 Emergency Medicine, Imam Abdulrahman Bin Faisal University, King Fahd University Hospital, Dammam, SAU; 3 Radiology, Imam Abdulrahman Bin Faisal University, King Fahd University Hospital, Dammam, SAU; 4 General Surgery, Imam Abdulrahman Bin Faisal University, King Fahd University Hospital, Dammam, SAU

**Keywords:** shock, septic, perforated, cancer, gastric

## Abstract

Perforated viscus is a fatal condition associated with a high mortality rate that necessitating immediate management. In gastric cancer, perforation is a relatively late rare presentation. In this study, we report a case of a 40-year-old male who presented with perforated gastric cancer. In the emergency department (ED), the provisional diagnosis was septic peritonitis and shock. However, upon exploratory laparotomy, pyloric tumor was detected metastasizing to the duodenum, liver, and porta hepatis.

## Introduction

Gastric carcinoma is one of the prevalent malignancies worldwide, having an increased incidence among the older population [1]. A systematic review was conducted to ascertain the risk factors for gastric cancer; the review discovered a total of 52 variables which were categorized into nine groups, demographics which included older age and male gender, low socioeconomic status, unhealthy diet and lifestyle, positive family history of gastric cancer, infections such as *Helicobacter pylori*, positive personal history of gastric surgery, esophageal cancer, or stomach polyp, occupational exposure to cement, mineral dust, chrome, and exposure to ionizing radiation [2]. Initially, gastric cancer patients typically experiencing persistent abdominal pain and weight loss [3]. Bloating, a palpable epigastric mass, nausea, and vomiting are all symptoms of gastric outlet obstruction, while cardiac involvement can lead to dysphagia, additionally, gastrointestinal bleeding may occur because of tumor ulceration [1,3]. Moreover, the liver, peritoneal surface, and distant lymph nodes are the most prevalent sites for metastasis [4]. Anorexia, jaundice, ascites, and hepatomegaly are some of the signs and symptoms linked to metastasis [1,3].

In contrary to previous studies that suggested gastric cancer does not invade the duodenum, it has been discovered that gastric cancer does invade the duodenum, which necessitating resection of the first part of it [5]. Diagnosis of gastric cancer can be delayed due to the absence of symptoms in the early stages [1]. Complete blood count (CBC), esophagogastroduodenoscopy (EGD), and upper GI series are some of the diagnostic tests available. Gastric carcinoma can be treated by gastrectomy, radiation, and chemotherapy after surgery to improve the chance of cure and extend survival rates [1].

Here, we report a case of undiagnosed gastric cancer that presented as gastric perforation and signs of septic shock. In the ED, the patient had a complaint of decreased oral intake and abdominal pain, even though there were no earlier visits that had indicated malignancy. During exploratory laparotomy, the patient was found to have gastric perforation and unresectable pyloric tumor metastasizing to the liver, duodenum, and porta hepatis.

## Case presentation

**Figure 1:**
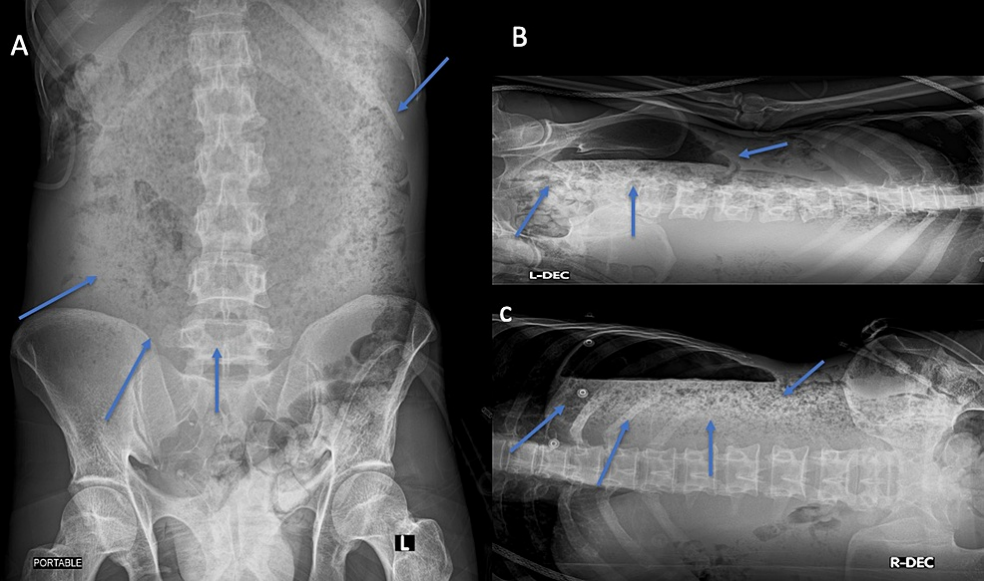
Supine (A) and lateral decubitus (B and C) abdominal x-rays show diffuse innumerable air foci (pointed by the blue arrows) mainly in the center of the abdomen (A) that corresponds to free air in the lateral decubitus (B and C) measuring around 15 x 5.8 cm (pointed by the blue arrows).

A 40-year-old Indian male had no known medical conditions. The patient had been experiencing loss of appetite, decreased oral intake, diffuse upper abdomen pain, and generalized body weakness for two weeks duration. On the clinical examination, the patient was semiconscious, had a hypotensive blood pressure of 70/60mmHg, and had tachycardia (pulse: 150/min). There was abdominal distention, generalized tenderness, and positive rebound tenderness which give the impression of generalized peritonitis. The patient was first stabilized in the ED by using the airway, breathing, and circulation (ABC) protocol for the management of a septic shock, which included a total of 3L of lactated Ringer’s solution. In addition, because the patient was unable to stand, supine left and right lateral decubitus abdominal x-rays were done (Figure 1). The images showed free air in the lateral decubitus measuring around 15x5.8 cm. Then, the patient was taken to the operating room (OR) after he was stabilized in the ED.

Upon exploration, pus was found filling the peritoneal cavity, and a large perforation in the greater curvature at the body of the stomach. There was a massive pyloric tumor that eroding the gastric serosa and spreading to underlying major vessels which make this tumor inoperable. Furthermore, the tumor was metastasized to the duodenum and liver. In addition, as the tumor was inoperable, a stomach stapling which is sometimes called vertical banded gastroplasty at greater curvature, gastrojejunostomy bypass, and insertion of distal feeding jejunostomy was done. 

Postoperatively, the patient went to the surgical intensive care unit (SICU) for continuous resuscitation and management. The patient spent 21 days in SICU due to the late manifestation of septic shock. Furthermore, while in SICU, the patient acquired several co-morbidities status and died from severe pneumonia and renal failure.

## Discussion

Perforated gastric carcinoma represents a rare complication [6]. Our patient presentation is consistent with the literature, which shows that viscus perforation is linked to the advanced stage of gastric cancer. However, it is linked to people who are older, while we report a case of a middle-aged patient. The preoperative malignancy diagnosis is rare, accounting for approximately 30% of cases [6]. Perforated viscus patients have sudden worsening of pain, and they are in obvious distress. Examination shows abdominal guarding and rebound tenderness. Investigation using an upright chest x-ray will show free air under the diaphragm in 80% of patient [7]. Two cases were reported of early perforated gastric cancer, one of 83-year-old female and the other 32-year-old male both had acute severe epigastric pain and they underwent upright chest x-ray which revealed air under diaphragm [8,9]. In this case, the patient was severely ill and had generalized abdominal pain. Furthermore, an upright chest x-ray was ordered to assess if there was any perforation, but the patient was unable to stand so supine, right and left lateral decubitus abdominal x-rays were ordered. 

In our case, the lateral decubitus abdominal x-ray was essential because it allowed the physician to identify the perforation in patients who were severely ill and cannot tolerate to stand for a chest x-ray. In addition, this will help in referring the patient to the OR as soon as possible. 

Perforated gastric carcinoma is treated by either one-stage gastrectomy or two-stage gastrectomy that includes initial treatment of peritonitis followed by elective gastrectomy [10]. Both reported cases were managed by gastrectomy [8,9]. Our patient was treated by stomach stapling at greater curvature, gastrojejunostomy bypass, and insertion of distal feeding jejunostomy because the tumor was inoperable. 

Perforated viscus is a life-threatening condition that accounts for high rates of mortality, regardless of the progress in both medical and surgical managements, overall mortality is 30% and reaches up to 70% in presence of diffuse peritonitis [11].

## Conclusions

In conclusion, the key message for emergency physicians is to consider perforated gastric cancer in their differential diagnosis list for patients who present having a septic shock owing to peritonitis. Furthermore, it is important to request lateral abdominal decubitus x-ray in patients who cannot stand when we suspect abdominal perforation because it will be helpful in early diagnosis and intervention. Despite the low incidence, physicians and surgeons should be aware of this possibility and arrange appropriate management plans based on the patient status.
